# Validity and reliability of the Hebrew version of the Brief Questionnaire of Olfactory Disorders (Brief-QOD) and the Self-Reported Mini Olfactory Questionnaire (Self-MOQ)

**DOI:** 10.1186/s41687-025-00961-7

**Published:** 2025-11-25

**Authors:** Tal Hefetz, Firas Kassem, Ameen Biadsee, Thomas Hummel, Ilan Blau, Dafna Gershnabel-Milk

**Affiliations:** 1 Department of Otolaryngology-Head and Neck Surgery, Meir Medical Center, Kfar Saba, 4428164 Israel; 2 School of Medicine, Faculty of Medical and Health Sciences, Tel Aviv University, Tel Aviv, 6997801 Israel; 3 Smell and Taste Clinic, Department of Otorhinolaryngology, Technical University of Dresden, Dresden, Germany

**Keywords:** Olfactory dysfunction, Brief-QOD, Self-MOQ, Patient reported outcome measures, Psychometric validation, Hebrew translation

## Abstract

**Objective:**

To translate and validate Hebrew versions of two patient-reported outcome measure questionnaires: the Self-Reported Mini Olfactory Questionnaire (Self-MOQ) and the Brief Questionnaire of Olfactory Disorders (Brief-QOD).

**Methods:**

A forward-backward translation process was conducted for both questionnaires. All participants rated their sense of smell using a general Visual Analog Scale (VAS) ranging from 0 (no dysfunction) to 10 (severe dysfunction). The patient group completed the questionnaires and the SNOT-22 questionnaire once. A control group of healthy participants completed the questionnaires twice to evaluate test-retest reliability. Subsets of both groups took the Sniffin’ Sticks test.

**Results:**

The translation process resulted in Hebrew versions deemed clear and culturally appropriate. A total of 91 individuals were enrolled in the control group and 62 in the patient group. The Hebrew versions of the Self-MOQ and Brief-QOD demonstrated high internal consistency (Cronbach’s α of 0.79–0.94 in the full sample) and overall test-retest reliability. The patient group had higher scores than the control group across all measures (*p* < 0.001). Logistic regression indicated that the Self-MOQ was the strongest predictor of group membership, while the Brief-QOD QOL and Visual Analog Scale components also significantly contributed. Receiver operating characteristic curve analysis identified an optimal Self-MOQ cutoff score of ≥ 2 for distinguishing patients from controls, with excellent accuracy (AUC = 0.97).

**Conclusions:**

The Hebrew Self-MOQ and Brief-QOD are reliable and valid tools for assessing olfactory dysfunction in the Hebrew-speaking population. Future research should evaluate questionnaire results after clinical interventions.

**Supplementary Information:**

The online version contains supplementary material available at 10.1186/s41687-025-00961-7.

## Introduction

Olfactory dysfunction (OD) increases with age, with a prevalence of more than 20% in the adult population [1]. OD affects wide aspects of daily life, particularly food intake (reduced enjoyment, decreased appetite, and cooking difficulties), safety (risk of eating spoiled food and failure to detect fire, smoke, or gas), personal hygiene, social interactions and work performance [2–5]. A reduced sense of smell can also serve as an early marker for neurodegenerative diseases like Parkinson’s and Alzheimer’s [6, 7]. Additionally, OD gained significant attention due to the COVID-19 pandemic, as it emerged as a hallmark symptom of the disease [8, 9]. Several studies investigated long-term changes in olfactory function among individuals affected by COVID-19, with reports of smell alterations persisting months after recovery from the acute phase of the disease [10, 11].

The diagnostic process for OD typically involves a combination of patient-reported outcome measures (PROMs), clinical history, physical examination, imaging studies, and psychophysical testing [12]. PROMs are frequently employed to evaluate the impact of OD on daily life, offering valuable insights into patients’ subjective perception of their condition. As one of the quickest and most straightforward methods for estimating olfactory function, PROMs play a meaningful role in the overall assessment. However, because subjective ratings may not always align with objective olfactory performance due to factors such as individual perception and psychological influences, they are best used in conjunction with psychophysical tests to ensure a more accurate and comprehensive evaluation [13].

Two well-established PROMs used to evaluate OD are the Self-Reported Mini Olfactory Questionnaire (Self-MOQ) and the Brief Questionnaire of Olfactory Disorders (Brief-QOD). The Self-MOQ is a concise screening tool designed to assess OD by capturing individuals’ self-perceived ability to smell in daily life. Its simplicity and brevity make it practical for use in both clinical and research settings [14]. The Brief-QOD is an abbreviated version of the original Questionnaire of Olfactory Disorders and is also widely applied in clinical and research contexts [15, 16], evaluating the perceived impact of olfactory dysfunction.

Efforts to adapt these questionnaires for non-English-speaking populations have led to translations of the Brief QOD into several languages, including Spanish, Italian, Arabic, French, and Malay. Similarly, the Self-MOQ has been translated into Arabic and Italian, broadening access to these tools and enabling cross-cultural comparisons and improved olfactory assessment [17–23].

Currently, validated Hebrew-language PROMs are lacking. To the best of our knowledge, the validity and reliability of Hebrew versions of these questionnaires have not been reported in the published literature. The present study translated and validated Hebrew versions of the Self-MOQ and the Brief-QOD to provide accurate, culturally-adapted tools for assessing OD.

## Materials and methods

### Ethics

The study was performed according to the ethical standards presented in the Declaration of Helsinki. It was approved by the Institutional Review Board of the medical center. Informed consent was obtained from all participants.

### Translation process

To ensure linguistic and cultural accuracy in the translation of the Self-MOQ and Brief-QOD from English to Hebrew, we employed a structured, forward-backward translation methodology. First, two individuals fluent in both English and Hebrew independently translated the questionnaires into Hebrew, ensuring that the meaning of each item was preserved while adapting it for Hebrew speakers. Following this, a committee of three physicians reviewed the translations, compared the versions, and consolidated them into a single optimal version. The Hebrew version was translated back to English by another physician who is fluent in both languages. Finally, the same committee reviewed the back-translation to verify its accuracy with the English version. This approach is well-aligned with the method for cross-cultural translation in questionnaire-based research [24].

### Population

Participants were recruited into two groups: a control group consisting of individuals who self-reported normal olfactory function, and a patient group comprising individuals who reported OD. All participants were recruited from the Department of Otorhinolaryngology at our institution, including the outpatient clinic, inpatient ward, and emergency department, between June/2023 to December/2024. To minimize confounding factors related to olfactory function, only individuals without any complaints related to smell, sinonasal conditions, or neurological symptoms were included in the control group. The patient group included individuals evaluated in the rhinology pre-operative clinic and the olfactory outpatient clinic. These patients were considered appropriate for assessing the reliability of OD assessment tools.

### Inclusion criteria

The inclusion criteria for both groups were: at least 18 years of age, able to provide signed informed consent indicating their willingness to participate in the study, and a native Hebrew speaker to ensure accurate comprehension of the questionnaires. Additionally, individuals with cognitive impairments that could interfere with their ability to complete the questionnaires reliably were excluded.

### Olfactory assessment

All participants were asked to rate their perceived olfactory function using a VAS ranging from 0 (no dysfunction) to 10 (severe dysfunction). They also completed the Hebrew-translated versions of the Self-MOQ and Brief-QOD questionnaires. The Self-MOQ is composed of five binary (yes/no) items, with each “yes” response assigned one point, resulting in a total score ranging from 0 to 5. The Brief-QOD includes three subdomains: Parosmia and QOL, assessed using a 4-point Likert scale (0 = Do not agree, 1 = Partially not agree, 2 = Partially agree, 3 = Agree), and a VAS section consisting of three questions rated from 0 to 10. All subdomain scores are summed to generate a total Brief-QOD score.

Participants in the control group completed the questionnaires on two occasions approximately one month apart, to assess test-retest reliability. A subset of participants from both the control and patient groups also completed the ODOFIN Sniffin’ Sticks Identification Test (Burghart Messtechnik GmbH, Holm, Germany) to allow comparison between subjective questionnaire responses and psychophysical olfactory performance. The patient group completed the translated questionnaires and the validated Hebrew version of the SNOT-22 questionnaire [25] to assess their sinonasal symptoms.

### Data analysis

Statistical analysis was conducted with SPSS version 29.0 and AMOS 30.0 (IBM Corp, Armonk, NY, USA). Differences between the control and patient groups were evaluated using independent samples t-tests for the questionnaire scores. Test-retest reliability for the control group was examined via paired-t test comparison, Pearson correlations between two questionnaires in the control group participants and further evaluated using a two-way random effects model with absolute agreement [ICC (2,1)]. Absolute reliability was assessed with the Standard Error of Measurement (SEM) and the Smallest Detectable Change (SDC), and Bland-Altman plots were generated to visualize agreement (see Supplementary Material). Pearson correlations were also performed between the scores of the Self-MOQ and the Brief-QOD. The internal consistency of the translated Self-MOQ and the Brief-QOD was assessed using Cronbach’s α. In addition to internal consistency analyses, we evaluated the structural validity of the Brief-QOD by performing confirmatory factor analysis (CFA) for the proposed three sub-domains structure (Parosmia, QOL, VAS). Model fit was assessed using χ²/df, CFI, TLI, and RMSEA indices. To assess the ability of different variables to discriminate between the control vs. patient groups, a logistic regression model with forward selection was employed. In this case, the model examined how well specific questionnaire scores could predict group membership. Receiver operating characteristic (ROC) curve analysis was performed to determine the Self-MOQ cutoff score for distinguishing between control and patient groups. A p-value of < 0.05 was considered significant.

## Results

**Fig. 1:**
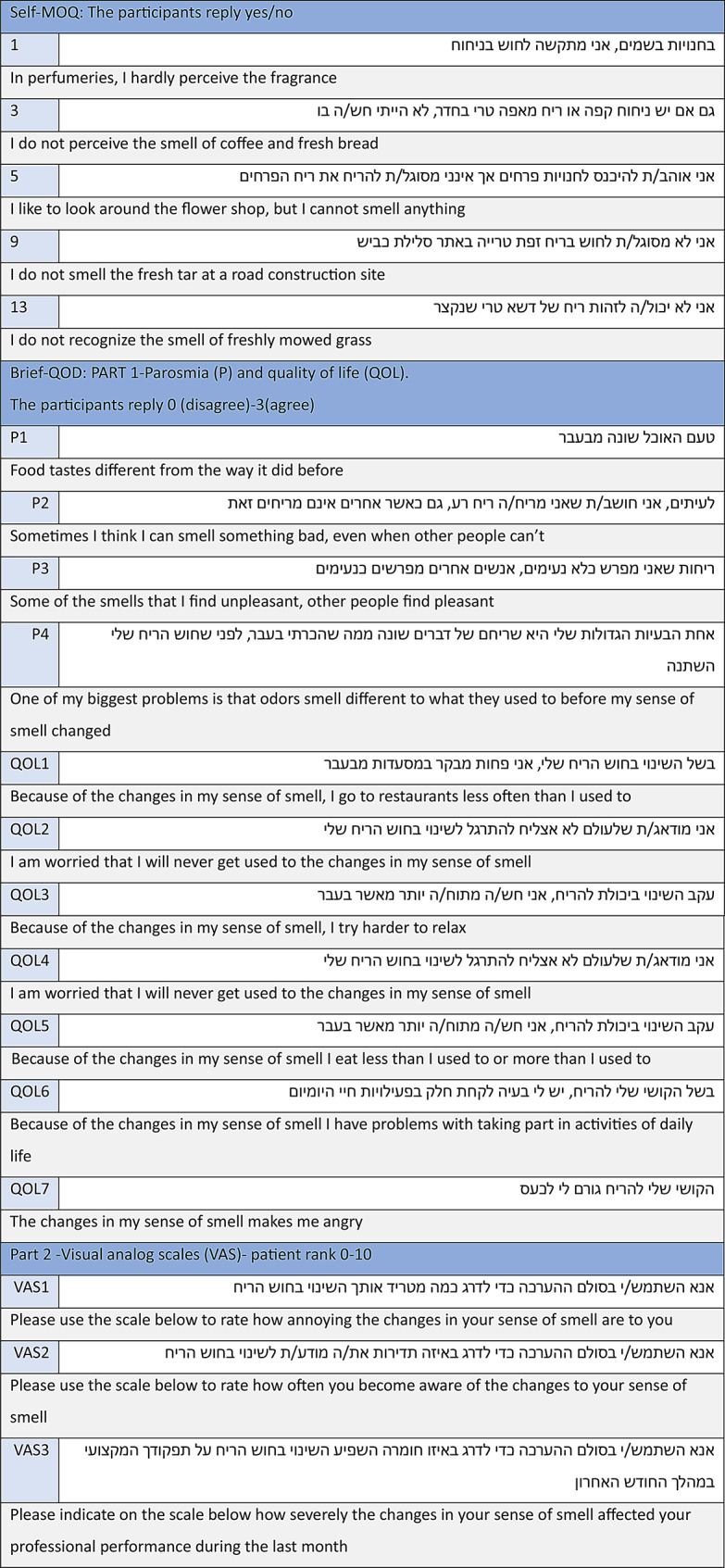
Self-MOQ and Brief-QOD Hebrew translation alongside the original English questionnaire

The final Hebrew versions of the Self-MOQ and Brief-QOD are presented in Fig. 1. A total of 153 participants were enrolled in the study. The control group consisted of 91 individuals (56 females and 35 males, mean age 42 years, SD = 13.1), who reported normal olfactory function. These participants completed the questionnaires on two separate occasions to assess test-retest reliability. Additionally, 23 participants from the control group underwent the Sniffin’ Sticks Identification Test. The patient group comprised 62 individuals (40 males and 22 females, mean age 45.6 years, SD = 16.3) experiencing OD, 12 of whom also completed the Sniffin’ Sticks test for comparison with their subjective assessments. Table 1 presents the demographic characteristics of the study participants.

**Table 1:** Demographic characteristics of study participants

Characteristic	Control (*n* = 91)	Patients (*n* = 62)	Statistical Test
Age, mean ± SD (y)	42.1 ± 13.1	45.6 ± 16.3	t-test: *p* = 0.16
Age Range (y)	19–76	19–78	
Sex			Chi-square: *p* = 0.003
Male, n (%)	35 (38.5%)	40 (64.5%)	
Female, n (%)	56 (61.5%)	22 (35.5%)	

### Group comparison (t-test)

Comparison of olfactory measures between the control and patient groups-including the general olfactory VAS, Self-MOQ, Brief-QOD, and Sniffin’ Sticks Identification Test-revealed consistent and statistically significant differences **(**Table 2**).** General olfactory VAS scores were significantly higher in the patient group (Mean = 8.13, SD = 1.97) compared to the control group (Mean = 0.41, SD = 0.82, *p* < 0.001), indicating a greater perceived burden of OD. Similarly, Self-MOQ scores were markedly elevated in the patient group (Mean = 3.71, SD = 1.52) relative to the control group (Mean = 0.29, SD = 0.64, *p* < 0.001). Patients also demonstrated higher scores across all subdomains of The Brief-QOD. In the Parosmia domain, the patient group scored higher (Mean = 6.34, SD = 3.52) than controls (Mean = 1.74, SD = 2.45 *p* < 0.001). For QOL, patient scores were also elevated (M = 9.45, SD = 6.76) compared to controls (Mean = 0.34, SD = 3.06, *p* < 0.001). In the VAS component of the Brief-QOD, the patient group again had higher scores (Mean = 20.39, SD = 6.78) versus the control group (Mean = 1.3, SD = 3.06, *p* < 0.001). Consequently, the total Brief-QOD score was substantially higher in the patient group (Mean = 36.18, SD = 14.00) than in the control group (Mean = 3.37, SD = 5.01, *p* < 0.001).

**Table 2:** Group comparisons of olfactory measures between control and patient participants

Variable	Control (*n* = 91)(SD)	Patient (*n* = 62) (SD)	t	*p*	Cohen’s d	95% CI of Cohen’s d	
						Lower	Upper
VAS	0.41 (0.82)	8.13 (1.97)	-29.19	< 0.001	-5.51	-6.20	-4.81
Self-MOQ Total	0.29 (0.64)	3.71 (1.52)	-16.77	< 0.001	-3.16	-3.64	-2.68
Brief QOD P Total	1.74 (2.45)	6.34 (3.52)	-8.94	< 0.001	-1.57	-1.94	-1.20
Brief QOL Total	0.34 (1.74)	9.45 (6.76)	-10.37	< 0.001	-2.02	-2.42	-1.63
Brief VAS Score	1.30 (3.06)	20.39 (6.78)	-20.78	< 0.001	-3.88	-4.43	-3.34
Brief Total Score	3.37 (5.01)	36.18 (14.00)	-17.70	< 0.001	-3.38	-3.88	-2.88
Sniffin sticks*	13.04 (1.49)	6.75 (3.02)	6.80	< 0.001	2.96	1.95	3.94

Note: CI, confidence interval. *Sniffing sticks test was completed by 23 members of the control group and 12 patients

Performance on the Sniffin’ Sticks Identification Test also reflected this pattern, as the patient group had significantly lower scores (Mean = 6.75, SD = 3.02) than the control group (Mean = 13.04, SD = 1.49), *p* < 0.001. Additionally, among the 57 participants who completed the SNOT-22 questionnaire, the mean score was 63.5 (SD = 14), indicating a high symptom burden consistent with severe sinonasal disease [26].

### Internal consistency of the self-MOQ and brief-QOD

Cronbach’s α coefficients were calculated for the entire sample in the first measurement and for the control group in the second **(**Table 3**)**. The results indicate high reliability across all measured variables for the first measurement (*n* = 153), with α = 0.90 for the Self-MOQ, 0.79 for the Brief-QOD Parosmia domain, 0.94 for QOL, 0.91 for VAS, and 0.80 for the total Brief-QOD. In the control group, second measurement Cronbach’s α values ranged from 0.50 to 0.86, reflecting moderate to high reliability and supporting the overall consistency of the Hebrew versions.

**Table 3:** Cronbach’s α values for internal consistency

Variable	Cronbach’s α Entire sample T1 (*n* = 153)	Cronbach’s α Healthy group only T2 (*n* = 91)
Self-MOQ	0.90	0.66
Brief-QOD P	0.79	0.70
Brief-QOD QOL	0.94	0.86
Brief-QOD VAS	0.91	0.64
Brief-QOD Total	0.80	0.50

α was considered reliable for values above 0.7

### Confirmatory factor analysis of the brief-QOD

CFA supported the expected three-factor solution, with all items loading significantly on their respective latent variables. Standardized factor loadings were generally high (0.75–0.93), except for two Parosmia items that showed weaker loadings (R² = 0.33–0.38). Model fit indices indicated suboptimal fit (χ²/df = 4.39, CFI = 0.87, TLI = 0.84, RMSEA = 0.15). Inter- factor correlations were substantial (*r* = 0.75–0.80).

### Discrimination between groups using logistic regression

Findings indicated that the Self-MOQ demonstrated the strongest discriminatory ability between the groups, with each one-point increase in the total score predicting a 3.82-fold increase in the likelihood of belonging to the patient group compared to the control group (*p* = 0.02). The Brief-QOD demonstrated partial discriminatory ability between the control and patient groups. The QOL and VAS components were significant predictors of group membership. Each one-point increase in their scores was associated with a 1.27-fold and 1.36-fold increase, respectively, in the probability of belonging to the patient group (*p* = 0.05 and *P* < 0.001, respectively).

### Test-retest reliability

In the control group, paired t-tests showed no significant differences between the first and second measurements of the General VAS, Brief-QOL, or Brief-VAS Score (*p* > 0.05). A minimal, though statistically significant differences, were observed in the Self-MOQ Total (mean difference of 0.12/5 points, *p* = 0.02), the Brief-QOD P domain (mean difference of 0.54/12 points, *p* < 0.001) and the Brief-Total (mean difference of 0.66/63 points, *p* < 0.001). Effect sizes represented by Cohen’s *d* values ranged from 0.00 to 0.37, indicating minimal change over time **(**Table 4**)**.

**Table 4:** Test-retest results of olfactory questionnaires in the control group

Variable	Mean Difference (SD)	t	*p*	Cohen’s d	95% CI of Cohen’s d		Pearson Correlation	*P*
					Lower	Upper		
General VAS	0.03 (1.05)	0.30	0.38	0.03	-0.17	0.24	0.40	< 0.001
Self-MOQ	0.12 (0.55)	2.08	0.02	0.22	0.01	0.43	0.59	< 0.001
Brief-QOD P	0.54 (1.46)	3.53	0.00	0.37	0.16	0.58	0.80	< 0.001
Brief-QOD QOL	0.00 (0.6)	0.00	0.50	0.00	-0.21	0.21	0.94	< 0.001
Brief-QOD VAS	0.12 (2.79)	0.41	0.34	0.04	-0.16	0.25	0.50	< 0.001
Brief-QOD Total	0.66 (3.63)	1.73	0.04	0.18	-0.03	0.39	0.71	< 0.001

Note: CI, confidence interval; VAS, visual analogue scale

Pearson correlations between two measurements of the questionnaires indicated that the Brief-QOD subscales demonstrated particularly strong test-retest reliability, with the highest correlations observed in the QOL (*r* = 0.94) and Parosmia (*r* = 0.80) domains. The Self-MOQ showed lower, yet significant, correlation (all *p* < 0.001). Consistent findings were obtained when evaluating test-retest reliability using intraclass correlation coefficients [ICC (2,1)], the Standard Error of Measurement (SEM), and the Smallest Detectable Change (SDC) (Table 5). The Brief-QOD QOL domain demonstrated excellent reliability (ICC = 0.96, SEM = 0.42, SDC = 1.16), and the Parosmia domain showed good reliability (ICC = 0.77, SEM = 1.08, SDC = 3.00). The overall Brief-QOD total score reached moderate reliability (ICC = 0.70, SEM = 2.58, SDC = 7.17). The Self-MOQ total score showed moderate reliability (ICC = 0.58, SEM = 0.40, SDC = 1.10). Reliability of the VAS scales was lower, with ICC values of 0.39 (general VAS) and 0.49 (Brief-QOD VAS). Bland-Altman plots are provided in the Supplementary Material.

**Table 5:** Test–retest reliability measures (ICC, SEM, SDC) in the control group

	ICC (2,1) [95% CI]	SEM (units)	SDC (units)	SDC (% of scale range)	Interpretation
GENRAL VAS	0.39 [0.20–0.55]	0.74	2.05	20.45%	Poor
SELF MOQ	0.58 [0.42–0.70]	0.40	1.10	22.01%	Moderate
BRIEF-QOD P	0.77 [0.65–0.85]	1.08	3.00	25.01%	Good
BRIEF-QOD QOL	0.96 [0.91–0.94]	0.42	1.16	5.54%	Excellent
BRIEF-QOD VAS	0.49 [0.32–0.63]	1.96	5.44	18.14%	Poor
BRIEF-QOD Total	0.70 [0.57–0.79]	2.58	7.17	11.37%	Moderate

### Inter-questionnaire correlations

Pearson correlation analyses between the two questionnaires (Table 6) indicated significant associations in the patient group. The Self-MOQ correlated moderately with the Brief-QOD VAS (*r* = 0.52, *p* < 0.001) and with the QOL subscale (*r* = 0.32, *p* < 0.05), while correlation with the Parosmia domain was weaker (*r* = 0.25, *p* < 0.05). The total Brief-QOD score correlated at *r* = 0.47 (*p* < 0.001). In addition, the Self-MOQ showed a moderate correlation with SNOT-22 results (*r* = 0.33, *p* < 0.05). In the control group, inter-questionnaire correlations were consistently weak to moderate across both assessments (baseline *r* = 0.32–0.36, follow-up *r* = 0.31–0.40; all *p* < 0.05), while the QOL domain did not reach significance (*r* = 0.01–0.06).

**Table 6:** Pearson correlations between the two questionnaires among the healthy and patient Groups

**Control group (** *n* ** = 91)**	**Brief-QOD ** *P* **-T1**	**Brief-QOD- QOL-T1**	**Brief-QOD VAS-T1**	**Brief-QOD Total-T1**
**Self-MOQ T1**	0.32**	0.01	0.33**	0.36***
**Control group (** *n* ** = 91)**	**Brief-QOD P-T2**	**Brief-QOD- QOL-T2**	**Brief-QOD VAS-T2**	**Brief-QOD Total-T2**
**Self-MOQ T2**	0.31**	0.06	0.40***	0.39***
**Patient group (** *n* ** = 62)**	**Brief-QOD P-T1**	**Brief-QOD- QOL-T1**	**Brief-QOD VAS-T1**	**Brief-QOD Total-T1**
**Self-MOQ T1**	0.25*	0.32*	0.52***	0.47***

*p < 0.05, **p < 0.01, ***P < 0.001

To note that patients completed the questionnaires 1 time only

### Receiver operating characteristic curve

**Fig. 2:**
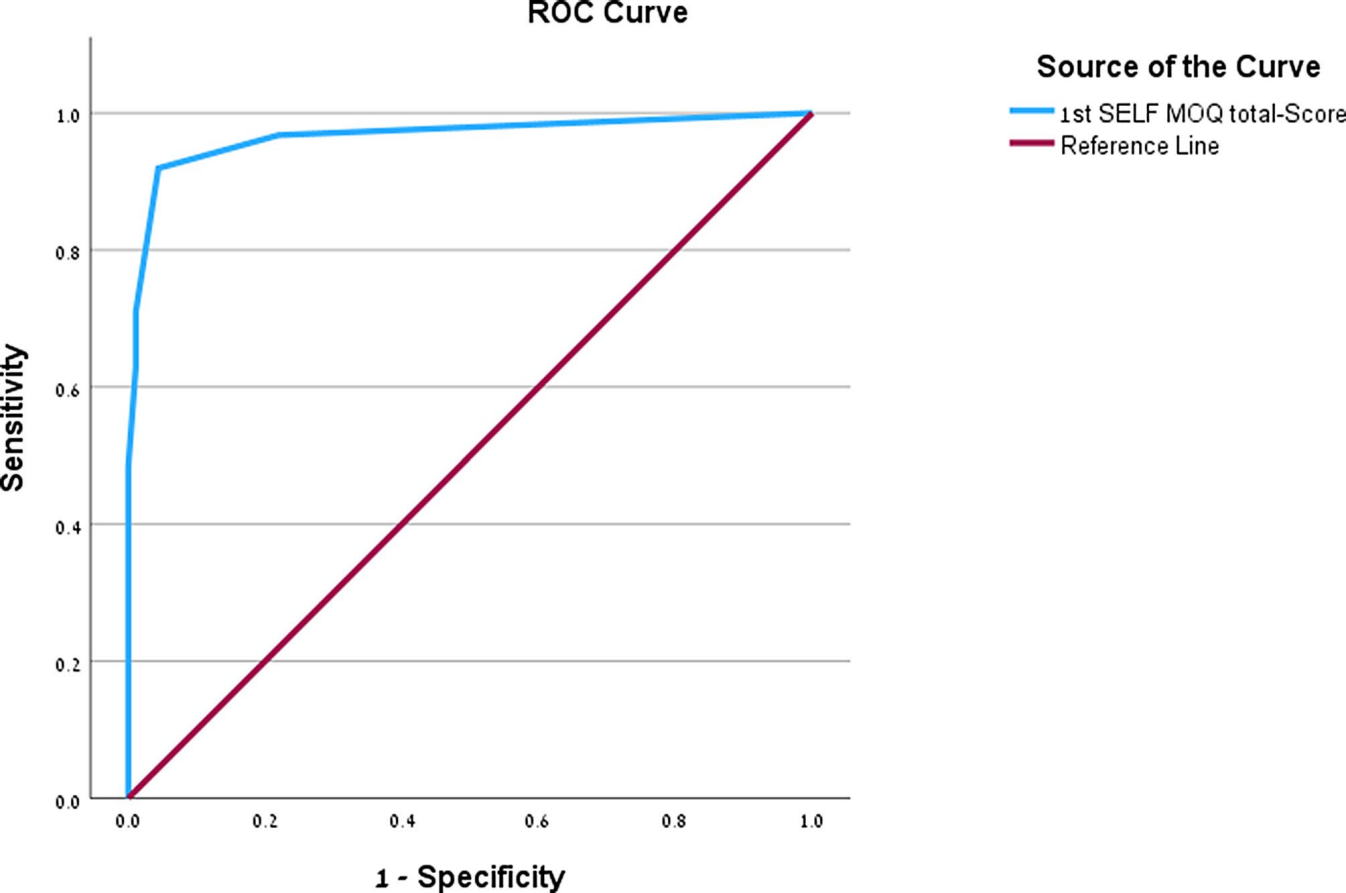
Receiver operating characteristic curve of the Hebrew Self-MOQ results: control vs. patient group. Area under the curve = 0.97

ROC analysis was performed to assess the ability of the Hebrew Self-MOQ to discriminate between patients and controls. The cutoff point yielding a balanced sensitivity (91.9%) and specificity (95.6%) was found to be a total Self-MOQ score of ≥ 2, with an area under the curve (AUC) of 0.97 (Fig. 2**)**. In the subgroup of patients and control participants who completed the Sniffin’ Sticks test (*n* = 35), a similar analysis yielded an identical cutoff of ≥ 2, with 100% sensitivity and 96% specificity, with an AUC of 1.0.

## Discussion

This study aimed to translate, culturally adapt, and validate Hebrew versions of two widely used PROMs: the Brief-QOD and the Self-MOQ, for use in the Israeli population. During the validation phase, all subjective olfactory measures (the Self-MOQ and the Brief-QOD, including its Parosmia, QOL, and VAS subscales) showed significant differences between the control and patient groups, supporting the sensitivity of these tools in distinguishing affected individuals. While only a subset of participants completed the Sniffin’ Sticks Identification Test, this psycho-physical assessment aligned with the self-reported data, demonstrating significantly reduced olfactory function in the patient group. These findings support the validity of both Hebrew-translated questionnaires. Test-retest reliability in the control group was demonstrated by non-significant paired t-tests in most questionnaire domains, while the small but statistically significant differences observed in the Self-MOQ total and Brief-QOD Parosmia subscales were of negligible magnitude. Pearson correlations ranged from moderate (*r* = 0.40) to very strong (*r* = 0.94). These findings support the stability of both questionnaires over repeated measurements. The internal consistency analysis measured using Cronbach’s α, indicated high reliability for both the Self-MOQ and the Brief-QOD in the full cohort at baseline, with all values exceeding the accepted threshold of 0.7. These findings align with previous research validating these instruments in other languages [16–19]. In contrast, coefficients in the control group at follow-up were lower (α = 0.50–0.86), most likely reflecting little variability within healthy participants.

The CFA results confirmed that the items of the Brief-QOD generally clustered in line with the theoretical three-subdomain structure, with most standardized factor loadings falling in the high range. However, two Parosmia items demonstrated weaker loadings, and the model fit indices did not reach conventional benchmarks for good fit. In addition, the correlations between latent factors were substantial, indicating overlap across domains.

These findings suggest that although the Brief-QOD broadly captures the intended constructs, certain domains, particularly the Parosmia subscale, may benefit from further refinement. Future studies could evaluate whether revision or reduction of specific items improves the structural validity of the instrument while maintaining its clinical relevance. The logistic regression analysis highlighted the Self-MOQ as the strongest predictor of group classification, indicating its particular usefulness in screening for OD. While the QOL and VAS subdomains of the Brief-QOD also significantly predicted group status, the Parosmia domain and total Brief-QOD score did not- contrary to expectations. This discrepancy may be attributed to the relatively small sample size and limiting statistical power. In addition, this difference may be explained by the primary purpose of the Brief-QOD, which is to assess the impact of OD on QOL, rather than diagnosing OD. The correlation between the Self-MOQ and the Brief-QOD were weak to moderate in both groups, with somewhat stronger associations in patients compared to controls. The Self-MOQ primarily reflects the severity of olfactory symptoms, whereas the Brief-QOD emphasizes the impact of these symptoms on quality of life. The modest overlap indicates that the instruments are related but not redundant, highlighting their complementary value in clinical assessment. ROC analysis showed the Self-MOQ had good discrimination abilities between patient and control groups with a cutoff threshold of ≥ 2 points in the full sample, lower than the threshold identified in the original validation study (3.5 for hyposmia) [14]. To note, The Italian and Arabic versions of the Self-MOQ did not establish a specific cutoff score for differentiating between normosmic and hyposmic individuals. Therefore, comparison between our work to other languages is not possible.

### Study limitations

This study had several limitations that should be considered when interpreting the findings. First, the patient group did not undergo a second measurement, limiting the ability to assess changes in follow-up or after treatment. In addition, while a subset of participants completed the Sniffin’ Sticks Identification Test, psychophysical olfactory testing was not conducted for the entire cohort. The small sample size can limit the power of the Brief-QOD to predict group classifications. Future research with larger groups might contribute to this issue. Another important limitation concerns the diversity of the Israeli population, which includes very large sectors that may not have strong proficiency in Hebrew, for example Arabic and Russian speaking patients. This point limits the applicability of the Hebrew version of the questionnaires among non-native Hebrew speakers. Although Russian versions of these tools have not yet been published, an Arabic version of both instruments has already been validated [23], enabling their use in parallel with the newly developed Hebrew version.

## Conclusion

This study successfully translated and validated the Hebrew versions of the Self-MOQ and Brief-QOD, demonstrating strong psychometric properties. Both tools showed high internal consistency in the full sample, and acceptable test-retest reliability among the control group. The Self-MOQ, as a diagnostic tool, showed superior ability to distinguish between individuals with or without OD. Future research should assess the effectiveness of these tools in large patient groups before and after clinical interventions.

## Supplementary Information

Below is the link to the electronic supplementary material.

Supplementary Material 1

## Data Availability

The datasets generated and analyzed during the current study are available from the corresponding author upon reasonable request.
