# Supplementary material for: Validity and reliability of the Hebrew version of the Brief Questionnaire of Olfactory Disorders (Brief-QOD) and the Self-Reported Mini Olfactory Questionnaire (Self-MOQ)
Source: J Patient Rep Outcomes. 2025 Nov 25;9:135. doi: 10.1186/s41687-025-00961-7 (PMC12647470; doi:10.1186/s41687-025-00961-7)

**Validity and Reliability of the Hebrew Brief Version of the Questionnaire of Olfactory Disorders (Brief-QOD) and the Self-reported Mini Olfactory Questionnaire (Self-MOQ)**

**Supplementary material**

**Translation and validation process:**

As part of the translation process, an informal check of the Hebrew versions of the Self-MOQ and Brief-QOD was carried out. A small number of volunteers, including both healthy participants and patients, were asked to complete the questionnaires and comment on clarity, wording, and ease of response. Based on their feedback, minor wording adjustments were introduced to improve readability and cultural appropriateness. The questionnaires were generally well understood and could be completed within a 3-5 minutes.

**Bland-Altman plots:**


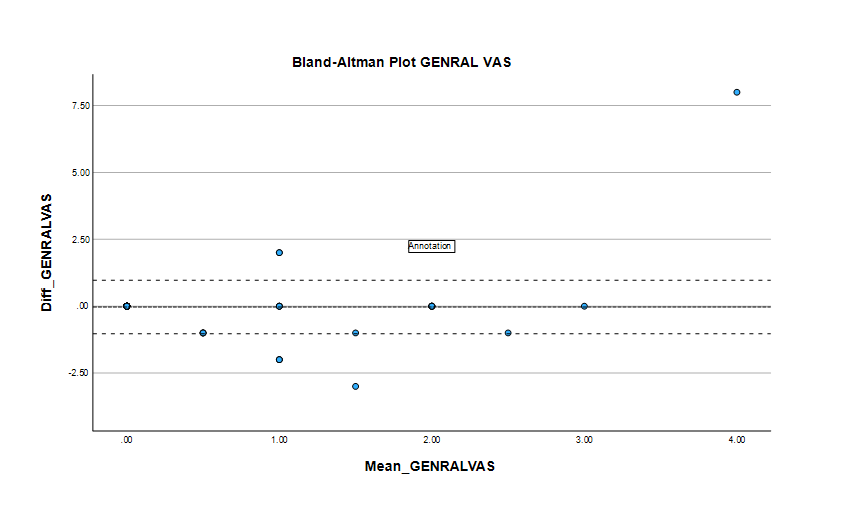


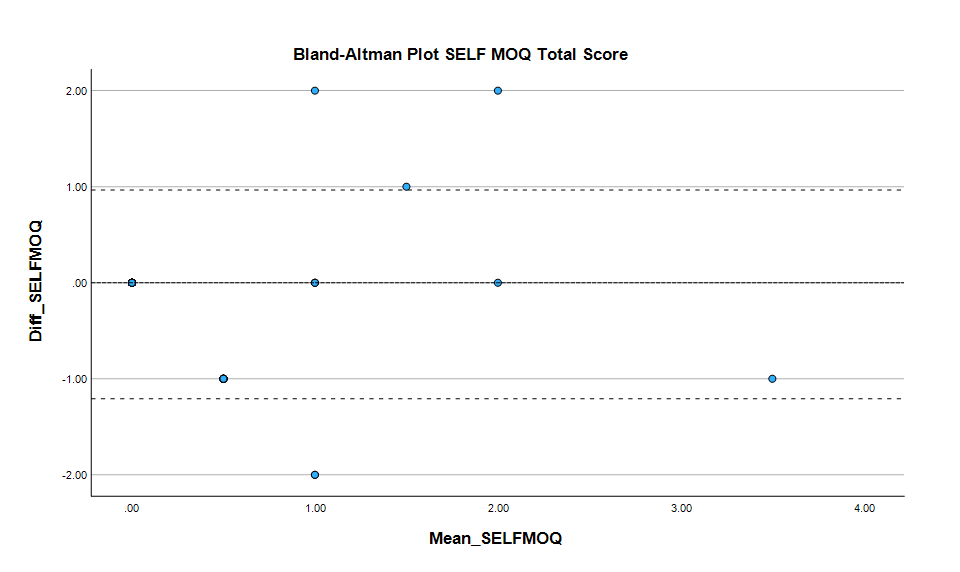


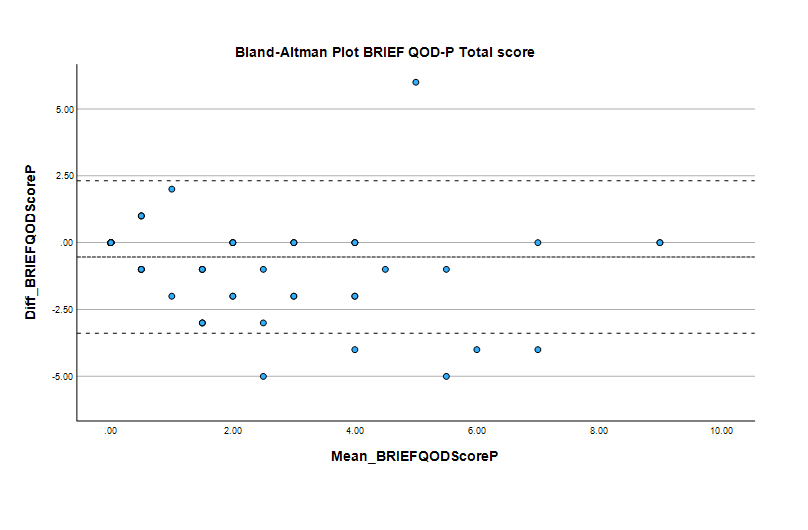


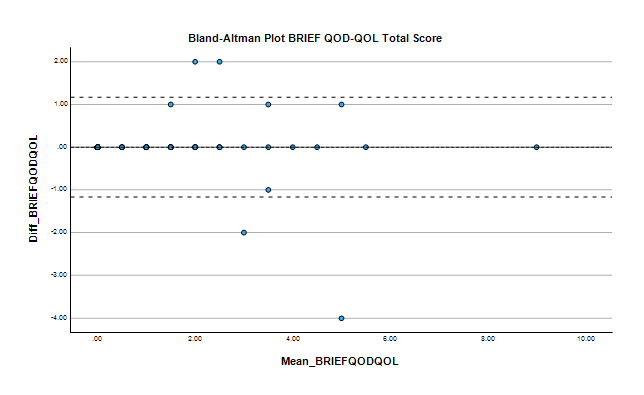


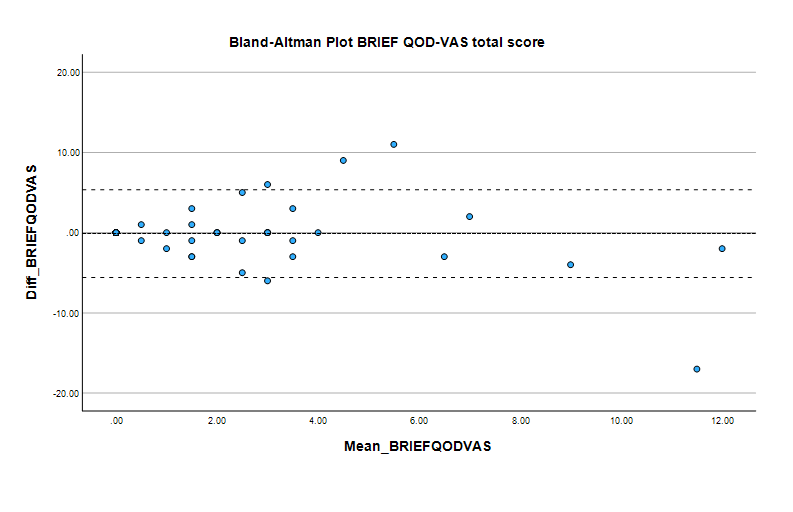


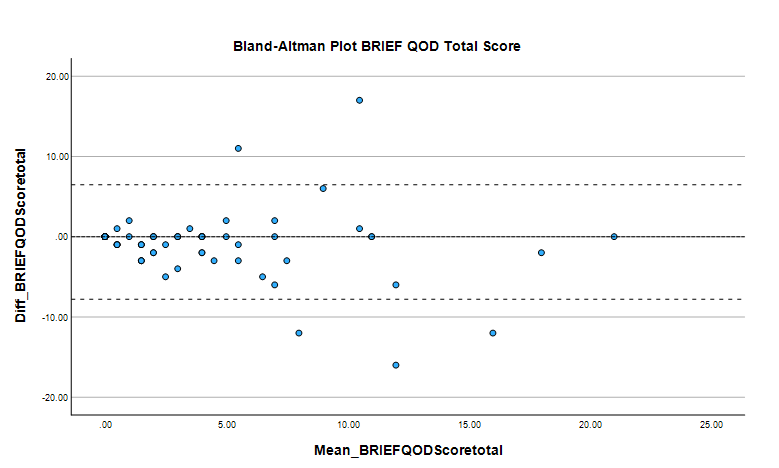

Supplement: Supplementary file 1 — Supplementary Material 1 [file 41687_2025_961_MOESM1_ESM.docx]
